# On the Prevention of Pitting in Confluent Small-Pox

**Published:** 1860-11

**Authors:** William Stokes

**Affiliations:** Regius Professor of Physic in the University of Dublin


*On the Prevention of Pitting in Confluent Small-Pox.* By WILLIAM STOKES, M. D., Regius Professor of Physic in the University of Dublin.—[The various modes previously employed, with a view of preventing pitting in cases of small-pox, may be thus enumerated: ]

1. The puncture of the pustules when matured.
2 The application of nitrate of silver.
3. The application of oil, or of the *linimentum calcis.*
4. The covering the face with a solution of gutta percha, with collodion, or with glycerine.

[The first of these modes is best adapted to a benign form of the disease, in which the pustules, though numerous, remain discrete for a longer period than in the severer cases. As to the second mode, Dr. Stokes has had no personal experience; it will be most suitable, like the last, in milder forms of the disease. The third form Dr. S. has tried, but does not consider either application of much value, though the linimentum calcis is preferable.]

During the past five years I have used gutta percha and collodion in a considerable number of cases. These, however, were not by any means examples of the worst form of the disease. In most of them the crust came off in large flakes or patches, composed obviously of the dried exudations and the covering material, and leaving the skin uninjured. To render this treatment effective, at least so far as the exclusion of the air is concerned, it is necessary to renew the application at intervals of from twelve to twenty-four hours; for the covering seems to be repeatedly broken up by the advance of the eruption and the swelling of the parts. Some patients are greatly distressed by the feeling of constriction caused by the coating of gutta purcha or collodion, and in general the treatment in question appears unadvisable where there is much vascularity, heat or swelling.

Looking at the frequency of pitting on the face, as compared with that of other parts of the surface, it is not easy to account for it, unless by referring to the fact that, while the rest of the surface is kept covered, and so not only comparatively excluded from the action of the air, but in a state of humidity, the integuments of the face remain in a dry and heated state—first, from the action of the external air, and next, from the increased vascular action. Hard and hot scabs are formed, and the ulcerative process makes its way downwards to a greater or less degree. Some have held that the liability of the face to markings was to be explained by anatomical considerations. However this may be, it will be found that in cases in which from an early period certain portions of the face have been kept protected from the action of the air, and in a permanently moist state, pitting does not occur. This may be seen in cases of sthenic confluent small-pox, where, with the view of preventing the adhesion of the eyelids, poultices have been used over the eyes. In such cases it will be often found that every part of the face is marked, except those over which the little poultices had extended.

The application of poultices over the face appears to me to be the surest mode of preventiug the consequent disfigurement. We should commence their use at the earliest period, and continue it to an advanced stage of the affection. In most cases they may be applied even over the nose, so as to cover the nostrils, for these passages are generally so obstructed as to be for the time useless to the patient. If the nostrils can be kept pervious by injections, the poultices need not be applied over their orifices.

If the chances of marking are in proportion to the activity of the cutaneous irritation, we may hold that this method should fulfil three important indications of treatment—

1. The exclusion of air;
2. The moderation of the local irritation; and,
3. The keeping of the parts in a permanently moist state, so as to prevent the drying and hardening of the scabs.

The value of this treatment, however, will, I fell convinced, be best seen in the inflammatory or sthenic form of the disease. The best poultice will be that formed of linseed meal, which should be spread on a soft material, such as French wadding, and covered with the gutta-purcha paper or oiled silk. I have never had occasion to repeat the adoption of this practice.

[Dr. Stokes sums up his paper with the following conclusions:]

1. That the chances of marking are much greater in the sthenic or inflammatory, than in the asthenic or typhoid confluent small-pox.

2. That considering the change in the character of disease, both essential and local, observed during late years, we may explain the greater frequency of marking in former times.

3. That in the typhoid forms of the disease the treatment of the surface by an artificial covering, such as gutta percha, or by glycerine, will often prove satisfactory.

4. That in the more active or non-typhoid forms, the use of constant poulticing, and of every other method that will lessen local inflammation, seems to be the best method of preventing the disfigurement of the face.—*Dub. Quarterly Journal*, p. 111.

